# Validation of Stable Reference Genes for RT-qPCR Normalization in *Oxycetonia jucunda* (Coleoptera: Scarabaeidae)

**DOI:** 10.3390/insects17010057

**Published:** 2026-01-01

**Authors:** Shi-Hang Zhao, Yang Yue, Rui-Tao Yu, Qi Gao, Jia-Qiang Zhao, Sheng-Ping Zhang, Nan Zhou, Guo-Liang Xu

**Affiliations:** 1 Shijiazhuang Institute of Pomology, Hebei Academy of Agriculture and Forestry Sciences, Shijiazhuang 050061, China; zhaoshih3@haafs.org (S.-H.Z.); wrightyu@haafs.org (R.-T.Y.); qigao977@haafs.org (Q.G.); jiaqiang_zhao@haafs.org (J.-Q.Z.); zspingyouxiang@haafs.org (S.-P.Z.); 2 Laboratory of Insect Collection, Shijiazhuang Institute of Pomology, Hebei Academy of Agriculture and Forestry Sciences, Shijiazhuang 050061, China; 3 College of Agriculture, Guangxi University, Nanning 530004, China; yueyang1014@163.com

**Keywords:** *Oxycetonia jucunda* Faldermann, reference gene, gene expression, RT-qPCR, normalization

## Abstract

The polyphagous pest *Oxycetonia jucunda* Faldermann can cause substantial damage to a range of economically important crops, with the adult beetles feeding directly on floral tissues and young leaves. RT-qPCR is widely used to analyze gene expression, for which the selection of stable reference genes is essential for enabling an accurate normalization of expression. However, no systematic evaluations of suitable reference genes for RT-qPCR analysis using different tissues of *O. jucunda* have been conducted. To assess their applicability as reliable normalization controls, we used five computational methods to examine the stability of seven potential reference genes (*GAPDH*, *EF1α*, *RPS3*, *RPS18*, *RPL18*, *RPS31*, and *UBC5A*) across six adult tissues, with three biological replicates per tissue. The findings revealed *RPS3* and *RPS31* to be the most stably expressed. This pair of reference genes was further validated by normalizing the expression of the odorant-binding protein 3 (*OBP3*) target gene. Our findings will provide important foundational data for the accurate analysis of functional gene expression in *O. jucunda*.

## Simple Summary

*Oxycetonia jucunda* Faldermann is a polyphagous pest that inflicts damage on a range of fruit tree species. The selection of an appropriate reference gene is essential for the reliable analysis of gene expression when using real-time quantitative polymerase chain reactions. This study aimed to identify stable internal controls by evaluating the stability of seven candidate reference genes across several *O. jucunda* tissues and assessing their suitability using five different algorithms. To verify the screening results, we examined the expression patterns of the odor-binding protein gene *OBP3*. The most stable reference genes identified using these analyses will provide a basis for further molecular studies on *O. jucunda.*

## 1. Introduction

*Oxycetonia jucunda* Faldermann (Coleoptera: Scarabaeidae) is a notable polyphagous agricultural pest, the host range of which encompasses more than 30 economically important crops, including species in the families Rosaceae and Vitaceae [1], with the adult beetles feeding directly on the floral organs and young leaves of fruit trees. Severe infestations can lead to complete defoliation, significantly compromising fruit production, yield, and tree vigor [2]. Although current pest control strategies are primarily dependent on chemical methods, the peak of adult beetle activity often coincides with the full bloom stage of crops, such as apples, thereby posing a significant risk of off-target effects on pollinator communities [3,4,5,6]. Consequently, researchers urgently need to develop more sustainable and effective management strategies for the control of this harmful species. Achieving this goal will necessitate a comprehensive understanding of the molecular mechanisms underlying key physiological processes in *O. jucunda*, which will provide a basis for the development of novel green precision control tools, such as RNAi-based pesticides [7,8,9]. The successful application of RNAi, however, critically depends on the prior identification of effective target genes through accurate gene expression profiling. However, molecular resources for *O. jucunda*, including validated reference genes for real-time quantitative polymerase chain reaction (RT-qPCR) normalization, are generally lacking, thereby limiting molecular studies on this species. The maturation of next-generation sequencing has made it feasible to systematically elucidate the genetic basis of these molecular mechanisms [10]. Such functional studies typically rely on two major approaches: quantitative gene expression analysis and targeted RNA interference [11,12], with RT-qPCR serving as an indispensable technique for gene expression analysis, given its high sensitivity and accuracy.

As the gold standard technique for gene expression analysis, the reliability of RT-qPCR data is highly dependent on the selection of appropriate reference genes [13,14,15,16,17,18,19]. Therefore, establishing stable reference genes is an essential prerequisite for basic gene function research and for the downstream identification and validation of candidate genes suitable for RNAi mediated pest control. In insect gene expression studies, systematic experimental variation primarily arises from a heterogeneity in sample preparation, fluctuations in RNA extraction efficiency, and biases during reverse transcription. To minimize such technical variability, performing normalization using stably expressed reference genes is thus necessary [20,21,22,23].

Traditionally used reference genes include members of the ribosomal protein (RP) family (*RPS*/*RPL*), glyceraldehyde-3-phosphate dehydrogenase (*GAPDH*), ubiquitin-conjugating enzyme (*UBC*), and elongation factor-1α (*EF1α*) [24]. To ensure reliable normalization, the expression of such reference genes should remain stable among different tissues, developmental stages, and experimental conditions [25]. However, substantial evidence indicates that the stability of reference gene expression is often characterized by pronounced species specificity and tissue dependence, and, as yet, no universally applicable reference genes suitable for normalization across all experimental conditions have been identified [14,19,26,27].

To date, no systematic validation of reference genes has been conducted for *O. jucunda*. This study addresses this critical knowledge gap by presenting the first comprehensive identification and evaluation of stable reference genes for RT-qPCR normalization in this species. Consequently, in this study, we sought to address the current lack of validated RT-qPCR normalization standards by identifying and evaluating the stability of reference gene expression across multiple tissues in *O. jucunda* using a multi-algorithm analytical framework. Specifically, we evaluated seven candidate reference genes (*GAPDH*, *EF1α*, *RPS3*, *RPS18*, *RPL18*, *RPS31*, and *UBC5A*) for stability of expression in different *O. jucunda* tissues using five algorithms (ΔCt method, BestKeeper, geNorm, NormFinder, and RefFinder), with the final selection validated by profiling expression patterns of the odorant-binding protein gene *OBP3* in *O. jucunda*. Based on our findings, we identified a set of reliable reference genes for further gene expression and molecular mechanistic studies on this species.

## 2. Materials and Methods

### 2.1. Insect Source

Specimens of adult *O. jucunda* were collected from an apple orchard in Pingyang Town, Fuping County, Baoding City, Hebei Province, and were subsequently reared for successive generations in an artificial climate chamber (26 ± 1 °C, 70% ± 5% relative humidity, and a 14:10 h Light:Dark photoperiod) at the Shijiazhuang Institute of Pomology, Hebei Academy of Agricultural and Forestry Sciences. The beetles were fed fresh apples.

### 2.2. Tissue Sample Collection

Tissue samples from the antennae, head, thorax, abdomen, legs, and wings of sexually mature F2 generation (10 days post-eclosion) adult *O. jucunda* beetles were obtained via dissection and separation. For each tissue, a biological replicate was constructed by pooling tissues collected from 10 different individuals (5 males and 5 females). The collected samples were immediately placed in 1.5 mL microcentrifuge tubes, rapidly frozen in liquid nitrogen, and thereafter stored long-term at −80 °C in an ultra-low temperature freezer to ensure RNA integrity for subsequent experiments.

### 2.3. RNA Extraction and cDNA Synthesis

Total RNA was extracted from the tissue sample using TRIzol Reagent (TransGen Biotech, Beijing, China), following the manufacturer’s protocol. Subsequently, the concentration and purity of RNA samples were determined using a NanoDrop 2000 spectrophotometer (Thermo Fisher Scientific, Waltham, MA, USA); all samples had A260/280 ratios between 1.8 and 2.0, indicating high purity. Using a 1 μg sample of the quantified RNA as a template, reverse transcription to yield first-strand cDNA was performed using a cDNA reverse transcription kit (TransGen Biotech, Beijing, China).

### 2.4. Quantitative Real-Time PCR Analysis

The candidate reference genes were initially selected from our unpublished transcriptome dataset of *O. jucunda*. Following functional annotation against public databases, including Nr and Swiss-Prot, the complete sequences of the following seven conventional reference genes were obtained: *GAPDH*, *EF1α*, *RPS3*, *RPS18*, *RPL18*, *RPS31*, and *UBC5A*. The target sequences were amplified via conventional PCR, and the resulting PCR amplicons were purified and commercially sequenced by Sangon Biotech (Shanghai, China). Gene-specific primers for real-time RT-qPCR were designed using Premier Primer 5.0 (Premier Biosoft; https://www.premierbiosoft.com/, accessed on 6 June 2025) (Table S1), and synthesis was performed by Sangon Biotech (Shanghai, China). Amplification was performed using an ABI 7500 Fast real-time PCR system (Thermo Fisher Scientific, Waltham, MA, USA) in conjunction with a Hieff qPCR SYBR Green Master Mix (TransGen Biotech, Beijing, China). Reaction mixtures (20 μL) contained 10 μL of SYBR Green qPCR premix, 0.4 μL of each of the forward (10 μM) and reverse (10 μM) primers, 1 μL of cDNA template, and 8.2 μL of nucleated acid enzyme water. The amplification program was as follows: an initial denaturation at 95 °C for 5 min, followed by 40 cycles of denaturation at 95 °C for 10 s and annealing/extension at 60 °C for 30 s. Confirmation of the specificity of the amplification product was based on melting curve analysis. For each tissue type, we used three biological replicates, each with three technical repetitions. To evaluate amplification efficiency, we constructed standard curves using eight-fold gradient dilutions of the respective cDNA samples, and PCR efficiency and associated regression coefficients (R^2^) were calculated from the slope of the curves.

### 2.5. Analysis of the Stability of Candidate Reference Genes

On the basis of the Ct values obtained for RT-qPCR, we conducted analyses of reference gene stability [28]. Initially, the ΔCt method used raw Cq values, and gene stability was evaluated by calculating the standard deviation (SD) of these values across all tissue samples, with a lower SD indicating greater stability. For geNorm and NormFinder analysis, the original Ct value was initially converted to the 2^−ΔCt^ form, for which ΔCt is the smallest Ct value of the gene in all samples minus the Ct value of each sample [29].

In the geNorm (version 2002) analysis, gene stability is expressed as the indicator M, with values below 0.7 considered stable [14]. The number of optimized reference genes required was determined using paired variation analysis (V_n_/V_n+1_), with a threshold of 0.15. A V_n_/V_n+1_ value lower than this value indicates that n reference genes are sufficient for standardization.

NormFinder (version 20) provides stability values that can be used to identify the optimal reference gene by evaluating intra- and inter-group gene mutations, with lower values corresponding to higher stability [30]. BestKeeper (version 1) comprehensively evaluates the stability of a gene based on the standard deviation (SD), variation coefficient, and correlation coefficient of the Ct values for each gene, with genes assigned an SD value greater than 1 considered unstable [31]. Finally, using the RefFinder online platform (https://blooge.cn/RefFinder/, access date: 10 August 2025), we integrated the evaluation results obtained using the aforementioned four methods to generate a comprehensive stability ranking for each candidate gene [32].

### 2.6. Validation of Reference Gene Stability

To validate the stability of reference genes, we profiled expression of the odorant-binding protein gene *OBP3*, with expression levels in different tissues from adults normalized to both the top- and bottom-ranked candidate genes, and calculated using the 2^−ΔΔCt^ method. The experiment was conducted using three biological replicates, each consisting of three technical replicates.

### 2.7. Statistical Analysis

Differences in gene expression among the assessed tissues were analyzed for significance using a one-way ANOVA with Tukey’s test (*p* < 0.05) in IBM SPSS Statistics 25.0 (IBM Corp., Armonk, NY, USA). All figures were generated using GraphPad Prism 10.1.2 (GraphPad Software, San Diego, CA, USA).

## 3. Results

### 3.1. Specificity and Amplification Efficiency of RT-qPCR Primers

Standard curve analysis confirmed that all applied primers were amplified with high efficiency, with individual values ranging from 95.5% to 108.6%, which is within the accepted optimal range (90–110%), and that the correlation coefficients (R^2^) for all assays exceeded 0.99 (Table 1). The specificity of amplification was verified by the presence of a single peak in the melting curves for each primer pair. All negative controls, including NTC and no-RT, yielded negative results. We also established the high inter-replicate reproducibility of the amplification profiles (Figure S1). These experimental and reporting steps were designed in accordance with key MIQE guidelines to ensure data robustness.

### 3.2. ΔCt Method

**Figure 1:**
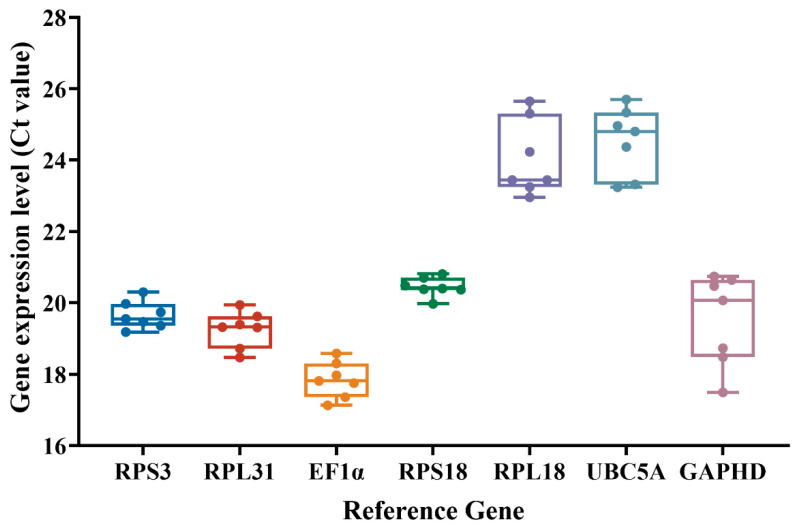
Expression of the candidate reference genes in different tissues obtained from *Oxycetonia jucunda*. The box plots show the median (center line), inter-quartile range (box), and range (whiskers). A more compact box and shorter whiskers indicate lower expression variability and higher stability.

The expression variation of the seven candidate reference genes across all tested tissues is visualized in Figure 1. Analysis of expression stability using the ΔCt method revealed that *RPS3* (0.59) and *RPS31* (0.62) were the most stably expressed genes, characterized by the least variation among samples, followed by *EF1α* (0.64) and *RPS18* (0.67), which showed moderate stability. Comparatively, *GAPDH* (1.22) and *UBC5A* (1.10) showed the most wide-ranging variability and were accordingly identified as the least stable.

### 3.3. GeNorm and NormFinder Analyses

**Figure 2:**
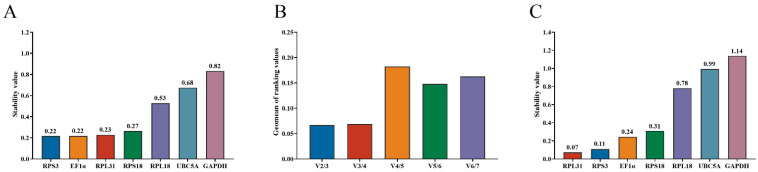
Stability analysis of candidate reference genes in different tissues of *O. jucunda*. (**A**) Expression stability ranking (M value) determined by geNorm. Lower M values indicate higher stability. (**B**) Determination of the optimal number of reference genes by geNorm pairwise variation (V_n_/V_n+1_) analysis. The dashed line indicates the 0.15 cutoff. (**C**) Stability ranking determined by NormFinder. Lower stability values indicate higher stability.

The stabilities of candidate reference gene expression were determined using geNorm with a threshold of M < 0.7 (Figure 2A). Among these genes, *EF1α* and *RPS3* (M = 0.219) had the highest stability, followed by *RPS31* (M = 0.229) and *RPS18* (M = 0.265). In contrast, *UBC5A* (M = 0.675) and *GAPDH* (M = 0.832) showed M values approaching or exceeding the threshold, indicating a lower stability. Pairwise variation V2/V3 values below the 0.15 threshold indicated that two reference genes were sufficient for reliable normalization (Figure 2B).

NormFinder analysis (Figure 2C) revealed that *RPS31*, which had the lowest stability value (0.074) among the candidate genes, had the highest expression stability, closely followed by *RPS3* (0.11) and *EF1α* (0.244). Conversely, we obtained notably higher stability values for *RPL18* (0.782), *UBC5A* (0.994), and *GAPDH* (1.139), with *GAPDH* exhibiting the greatest variability. These findings thus provided evidence that *RPS31* and *RPS3* were the most suitable genes for reference purposes in this experimental system, whereas *GAPDH* exhibited relatively poor stability.

### 3.4. BestKeeper Analysis

BestKeeper can be used to assess gene stability based on the standard deviation (SD) of Ct values (Table 2), with lower SD values indicating a higher stability of expression. According to the BestKeeper criterion, any gene with an SD greater than 1 is considered unstable, and we accordingly identified *RPS18* and *RPS3* as the genes exhibiting the highest expression stability among the assessed *O. jucunda* tissues.

### 3.5. RefFinder Analysis

Given that the algorithms geNorm, NormFinder, the ΔCt method, and BestKeeper employ distinct principles to evaluate gene stability, we utilized the RefFinder platform to integrate the respective results, thereby providing a consensus ranking of stability based on the geometric mean of the individual algorithm rankings (Table 3). Collectively, our analyses identified *RPS3* and *RPS31* as the most stable reference genes, as these two genes were consistently ranked among the top three most stable genes using all four algorithms. Conversely, *UBC5A* and *GAPDH* received the lowest comprehensive rankings, indicating that experimental conditions had a notable influence on their expression, hence accounting for the poor stability.

### 3.6. Validation of the Stability of Reference Genes

**Figure 3:**
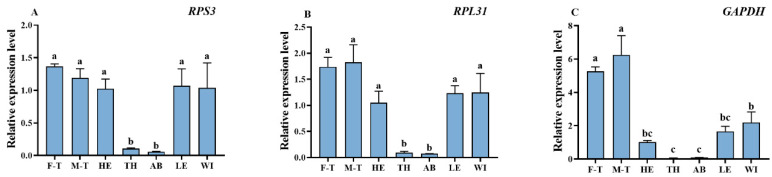
Relative expression of *OBP3* in *Oxycetonia jucunda* tissues normalized with different reference genes. Different lowercase letters above bars indicate statistically significant differences between the groups (*p* < 0.05). Tissue abbreviations: F-T, female tentacles; M-T, male tentacles; HE, heads (antennae removed); TH, thorax; AB, abdomen; LE, legs; WI, wings.

To assess the influence of reference gene selection, we compared the normalized expression of a target gene (*OBP3*) using the most and least stable gene candidates. The results revealed that when *RPS3* and *RPS31* were used as internal references, the expression trends of *OBP3* across different tissues were highly consistent. Conversely, using a less stably expressed gene, *GAPDH*, resulted in significant deviations in its expression patterns (Figure 3). These comparisons accordingly revealed that using either *RPS3* or *RPS31* individually can effectively minimize technical variation. In contrast, the inherent instability of *GAPDH* introduces substantial normalization bias.

## 4. Discussion

Although RT-qPCR is a fundamental method for assessing gene expression, the precision of the results depends on the use of stable reference genes to normalize the data. [21,33,34]. Extensive evidence indicates that reference gene expression can vary considerably across experimental conditions, including within the same species, and, at present, no universally applicable reference genes have been identified [35,36]. Consequently, to prevent analytical bias, candidate reference genes need to be rigorously validated under specific experimental conditions. Hence, seven candidate reference genes (*GAPDH*, *EF1α*, *RPS3*, *RPS18*, *RPL18*, *RPS31*, and *UBC5A*) were selected, the stability of expression of which was systematically analyzed in different tissues of *O. jucunda*, and on the basis of a comprehensive evaluation using four distinct algorithms, we established a set of optimal reference genes for multiple tissue expression analysis.

Among the assessed candidates, ribosomal protein genes, which play pivotal roles in cellular metabolism and growth regulation, are characterized by highly conserved sequences and functions among different species. Given their evolutionary stability, these genes have been widely adopted as internal reference genes across a range of insect studies [37,38,39]. For example, *RPS18* has been shown to exhibit stable inter-tissue expression in Gynaephora qinghaiensis [37], and RPS15 has been found to exhibit consistent normalization performance in the larval tissues of Helicoverpa armigera across different developmental stages [40]. Similarly, throughout the developmental cycle of *Plutella xylostella*, *RPS13* and *RPS23* are highly stable [41]. Consistent with these reports, *RPS3* and *RPS31* were identified as the most stable reference genes across the tested tissues of *O. jucunda*, supporting the utility of ribosomal protein genes for normalization. The specific superior stability of *RPS3* and *RPS31* among the RP genes assessed may be attributed to their fundamental roles in ribosome biogenesis and function. Notably, *RPS3* was consistently ranked among the top two most stable genes in the multiple algorithmic evaluations we performed. These findings thus reinforce the applicability of RP genes as reliable reference genes in *O. jucunda* and are consistent with evidence supporting the use of RPS or RPL genes for expression normalization in insects, thereby highlighting their broad utility in gene expression studies [42,43,44,45].

The *GAPDH*-encoded enzyme catalyzes the reversible oxidation and phosphorylation of glyceraldehyde-3-phosphate to 1,3-bisphosphoglycerate, a central step in both glycolysis and gluconeogenesis [46]. Given its essential function in core carbon metabolism, *GAPDH* is constitutively expressed at high levels among a wide range of tissues and cell types, and, as such, researchers often assume that the expression of this gene remains stable under different experimental conditions. Consequently, researchers widely employ it as a reference gene for normalizing quantitative data in molecular biology [47]. However, the validity of this assumption is questionable, as substantial evidence indicates that *GAPDH* expression is readily regulated by a range of physiological and pathological stimuli. Such regulation accordingly introduces substantial bias into gene expression analysis, thereby compromising the accuracy and interpretability of the results obtained [47,48]. Indeed, *GAPDH* was found to be unstable for normalizing expression across multiple tissue types in *O. jucunda*, consistently ranked as the least stable candidate by all algorithms and the RefFinder analysis. Validation using *OBP3* expression profiles corroborated this finding, confirming that *GAPDH* was the most unstable gene among those evaluated, consistent with findings reported for other species, including Aquatica leii, Bactrocera dorsalis, and Pandora neoaphidis, in which *GAPDH* is characterized by similarly unstable expression [49,50,51].

To ensure experimental reliability, we evaluated the stability of reference genes using four established algorithms (ΔCT, geNorm, NormFinder, and BestKeeper). Whereas the findings of ΔCT and geNorm analyses identified *RPS3* as the most stable, *RPS18* performed best in the BestKeeper analysis, and *RPS31* and *EF1α* ranked highest with NormFinder. This discrepancy in rankings can be ascribed to differences in the mathematical principles underlying the operation of these algorithms, and is a commonly observed phenomenon in such studies [45,51,52,53,54]. Notably, the geNorm algorithm is particularly sensitive to coregulation among candidate genes, as it ranks stability based on pairwise expression correlation. This can introduce a bias favoring genes with similar expression patterns, even if those patterns are not constitutively stable across conditions. To mitigate this limitation and obtain a robust consensus, we integrated the results from all four methods using RefFinder, with the consolidated ranking considered to serve as the definitive assessment of reference gene stability.

Currently, neither genomic nor transcriptomic data are available for *O. jucunda*. The identification of stable reference genes (*RPS3* and *RPS31*) in *O. jucunda* directly establishes the essential foundation for quantifying gene expression, which is a critical step in RNAi pest control. However, it will be necessary to further validate the stability of these candidate reference genes across a broader range of biological conditions, including at different stages of development and in response to environmental stresses, to establish a more robust and universal qPCR normalization framework for functional gene studies in this pest.

## 5. Conclusions

This study represents the first systematic evaluation of reference genes in *O. jucunda* based on transcriptomic data. Based on our comprehensive evaluation of seven candidate genes using five established computational methods, *RPS3* and *RPS31* were identified as the most stable reference genes across the assessed tissues. These findings will facilitate reliable reference gene application and provide a robust data foundation for future functional gene studies in this species.

## Figures and Tables

**Table 1:** Amplification efficiencies of the seven candidate reference genes in *Oxycetonia jucunda* identified by RT-qPCR.

Gene	Efficiency (%)	R^2^
*RPL18*	108.6	0.991
*RPS31*	107.3	0.997
*UBC5A*	106.1	0.997
*GAPDH*	95.6	0.995
*EF-1α*	95.5	0.998
*RPS3*	98.6	0.998
*RPS18*	96.5	0.998

**Table 2:** Results of evaluations of the stability of reference gene expression in different tissues of adult *Oxycetonia jucunda* using BestKeeper.

Parameter	*GAPDH*	*EF1α*	*RPS3*	*RPS18*	*UBC5A*	*RPL18*	*RPS31*
std dev [+/− CP]	1.1	0.38	0.3	0.19	0.76	0.88	0.38
CV [% CP]	5.64	2.12	1.53	0.92	3.1	3.64	1.95
*p*-value	0.056	0.02	0.001	0.045	0.464	0.041	0.006

**Table 3:** Comparative ranking of the stability of candidate reference genes.

Method	1	2	3	4	5	6	7
Delta CT	*RPS3*	*RPS31*	*EF1α*	*RPS18*	*RPL18*	*UBC5A*	*GAPDH*
BestKeeper	*RPS18*	*RPS3*	*RPS31*	*EF1α*	*UBC5A*	*RPL18*	*GAPDH*
Normfinder	*RPS31*	*RPS3*	*EF1α*	*RPS18*	*RPL18*	*UBC5A*	*GAPDH*
Genorm	*EF1α*|*RPS3*		*RPS31*	*RPS18*	*RPL18*	*UBC5A*	*GAPDH*
OVERALL	*RPS3*	*RPS31*	*EF1α*	*RPS18*	*RPL18*	*UBC5A*	*GAPDH*

## Data Availability

The original contributions presented in this study are included in the article/Supplementary Materials. Further inquiries can be directed to the corresponding authors.

## Supplementary Materials

The following supporting information can be downloaded at: https://www.mdpi.com/article/10.3390/insects17010057/s1, Table S1: Primers of reference genes in *O. jucunda*. Figure S1: Melting curve analysis of seven candidate reference genes.
