# Supplementary material for: Validation of Stable Reference Genes for RT-qPCR Normalization in Oxycetonia jucunda (Coleoptera: Scarabaeidae)
Source: Insects. 2026 Jan 1;17(1):57. doi: 10.3390/insects17010057 (PMC12842091; doi:10.3390/insects17010057)
Supplement: Supplementary file 1 [file insects-17-00057-s001.zip › insects-4039815-supplementary.pdf]

Table S1 Primers of reference genes in *O. jucunda*.

Gene Name	Primer Sequence (5'-3')
RPL18	F: AGAAAATGCTTTAGTATCCCA<br>R: GCTTTATCTGCCCCACC
RpL31	F: ATGGCGAAAACCAAGGA<br>R: CCGAACTCTAATACGGAAAG
UBC5A	F: ACGAACCAGCCGAAAATA<br>R: TGACCAATAGACCACAAAGAG
GAPDH	F: CGGAATCGTTGAGGGC<br>R: GGGAATTATGTTTTGGGCT
EF-1α	F: CCTTGCTGGCTTTCACA<br>R: CGACGATACCTCCTTTTTG
Rps3	F: GTAACCCCAACACGCAC<br>R: GATAGCACACAAACCCCTC
Rps18	F: TCCCTGACTGGTTCTTGA<br>R: GTTAGCTGACTGTATTTGCC
Obp3	F: AGTGTTTCTCCAGATGCTTT<br>R: CTTTTTCACGGTTGTGTTC

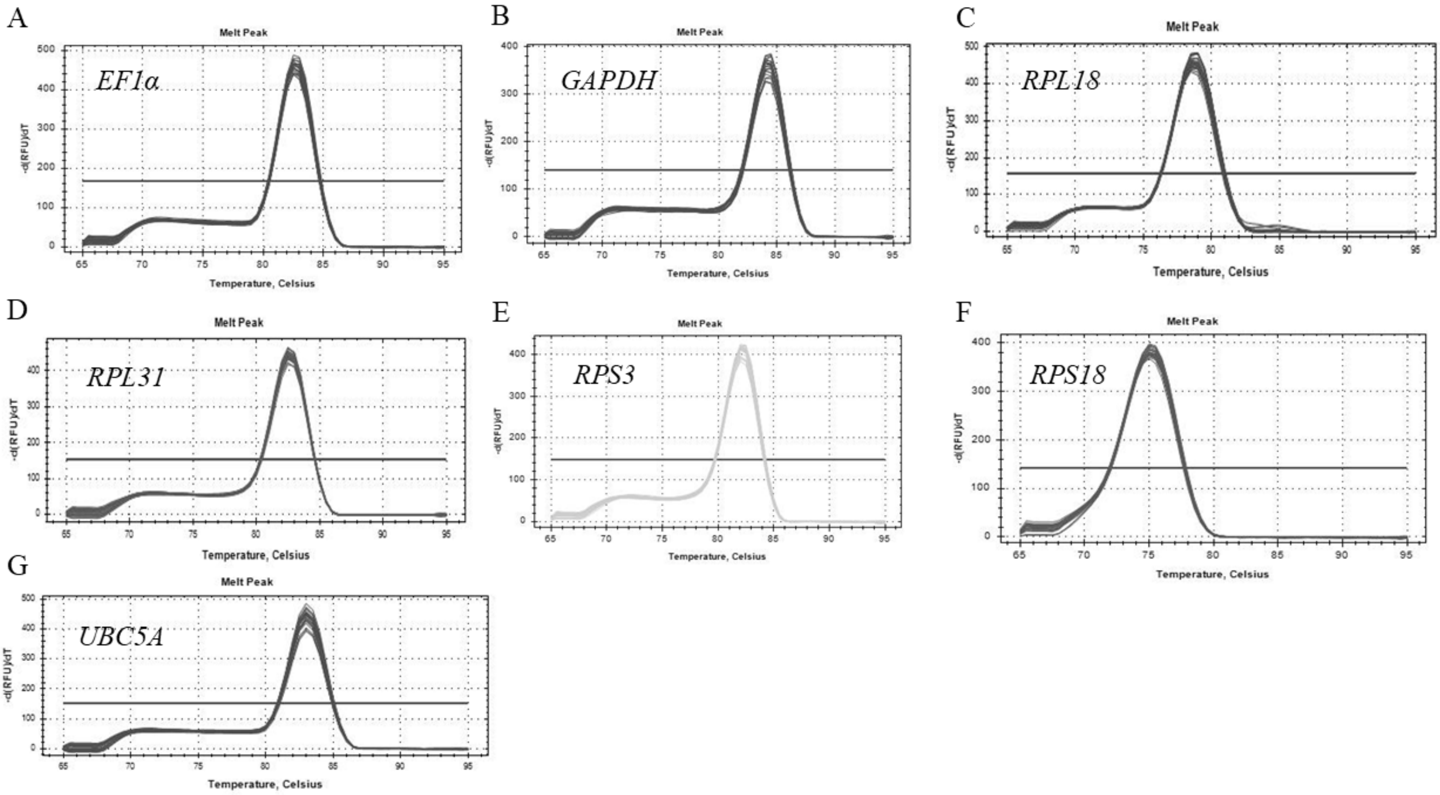

Figure S1 Melting curve analysis of seven candidate reference genes.
